# Thenar Compartment Syndrome: What If a Compartment Pressure Measuring Device is Absent?

**DOI:** 10.7759/cureus.2197

**Published:** 2018-02-16

**Authors:** Aydin Budeyri, Mehmet C Cankus, Gökhan Meric, Gökhan B Sever

**Affiliations:** 1 Department of Orthopaedics and Traumatology, SANKO University, Faculty of Medicine, Gaziantep, Turkey; 2 Department of Orthopaedics and Traumatology, Balıkesir University, Medical Faculty

**Keywords:** pressure monitoring device, thenar compartment syndrome, compartment syndrome of the hand, hand injury, anesthesia machine compartment pressure measurement, microsurgery, compartment pressure measurement, pressure monitorized compartment release, compartment syndrome, compartment pressure

## Abstract

Compartment syndrome (CS) is a threatening condition characterized by excessive tissue pressure accumulation associated with acute trauma. Compartment syndrome causes a significant reduction in blood flow with subsequent muscle and nerve ischemic necrosis. Recently, reports have described the importance of intramuscular pressure measurements as a basis for CS diagnosis. Unfortunately, the measuring devices that were utilized produced results with unsatisfactory reliability, making a diagnosis and subsequent treatment challenging. Here, we report the use of an anesthesia pressure monitoring device with greater precision for pressure measurements, as well as real-time monitoring of intraoperative compartment pressure decompression efficacy. This device enabled the accurate diagnosis and rapid treatment of a thenar compartment syndrome (TCS) in the left hand of a diabetic female in an emergency setting. She presented extreme pain in the thumb flexion-extension (FE). Her condition was complicated by diabetic cellulitis, primarily of Staphylococcus aureus. Consequently, successful microsurgery in the thenar space, together with debridement, resulted in remarkable pain relief during FE of the thumb metacarpophalangeal (MCP) and interphalangeal (IP) joints, as well as the disappearance of the infection by Day 10. Subsequent one- to two-year follow-up assessments revealed marked recovery.

## Introduction

Until recently, it has been unclear if there was any correlation between the thenar compartment and abrupt traumatic injury of the hand [1]. Now emerging reports indicate the existence of thenar compartment syndrome as a result of severe crush injury to the palm [1]. In recent years, it has been difficult to obtain precise measurements of compartment pressure to aid accurate diagnosis of compartment syndrome conditions [1-5], particularly in emergency situations. Thus, current reports focus on comparing the efficiency and reproducibility of several pressure-measuring device designs in an attempt to search for the best device and solve this problem but not ever for thenar compartment [1-6]. This study aimed to investigate the effectiveness of utilizing a modified anesthesia machine with an arterial pressure line transducer as a real-time compartment pressure monitoring device in a case-oriented study of a 45-year-old female in a neglected emergency situation. Pressure decompression efficacy recordings were measured continuously to enable diagnosis and the relief of compartment pressures in the left hand and subsequent microsurgical compartment decompression in this emergency situation. Subsequently, we show that short- and long-term pain and performance assessments on the tenth day and first two years demonstrated a gradual restoration of the left hand.

## Case presentation

Case illustration

A 45-year-old diabetic woman presented in the emergency care unit with a tender left thumb, as well as limited range of motion, tight swelling, erythema, and paresthesia in her left dominant-hand. She claimed a hand contusion trauma under a table occurring seven days prior to being presented to our clinic. Without any wounds on her hand, she was diagnosed physically and radiologically to have a soft tissue contusion without any fractures. A thumb spica resting cast had been applied by another clinic to the injured hand. No elevation or swelling-prevention information, or any type of advice had been given to her. Neglected thenar compartment syndrome, superimposed with diabetic cellulitis of the hand, was diagnosed when she was admitted to our clinic.

Diagnosis and treatment procedures

Physical Observations

**Figure 1:**
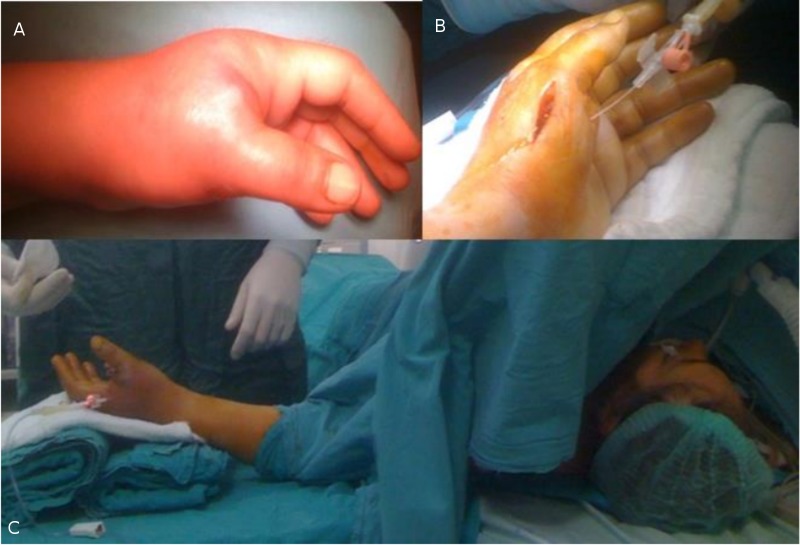
Photographs of the left hand Photographs of the left hand displaying: A) Extensive post-cast swelling without a base fracture of the soft tissue of the left thumb; B) A microsurgical longitudinal incision made on the radial side of the left-hand first dorsal interosseous compartment for catheter insertion, pressure measurements, and to allow decompression; C) Pressure measurements and monitoring in the left thenar and first dorsal interosseous metacarpal compartments during decompression.

Preoperatively, extreme pain during the flexion-extension of the thumb metacarpophalangeal (MCP) and interphalangeal (IP) joints was present. The physical appearance of the thumb and thenar area showed diffuse and tight swelling, coldness at the distal part of the thumb, an inadequately managed high level of pain, extreme pain with passive flexion and extension, and numbness manifesting at the thenar side of the thumb (Figure 1A).

Pressure Monitorization Workup and Surgical Decompression

To guarantee an accurate diagnosis of TCS, determining the compartment pressure was required [1]. This measurement provided the basis for treatment by compartment decompression. The anesthesia machine arterial pressure line transducer (AMAPLT) is a newly designed piece of equipment, based on specific modifications from previously reported devices [1-6]. We used this new device for compartment pressure measurements (CPM) for accuracy in an emergency situation. The equipment consists of many parts, including an anesthesia unit (which put the patient to sleep for pain relief during the microsurgery) hooked up to an arterial catheter and transducers, which in turn are connected to monitors. According to the emergency conditions, we did not have access to a slit catheter, so we created our own, which was of slit-like fashion, using a 20-gauge needle connected to an angiocatheter at one end. The other end of the catheter was connected to the arterial pressure transducer set at heart level. The transducer cable was connected to a monitor for pressure recording and display. A longitudinal 2.0 - 2.5 cm incision was made with a #15 sterile blade on the radial side of the left-hand first metacarpal to decompress both the thenar and the first and second intermetacarpal compartments. The angiocatheter, with the attached needle, aided the introduction of the catheter through the anterior-posterior wall of the thenar area for pressure measurements and monitoring. After enough pressure had been released, a Prolene® suture (5.0) (Ethicon, Inc., Cornelia, GA) was attached to the distal tip of the microcatheter and looped through the passage. CPM was constantly monitored in real-time to provide effective pressure and decompression information during the course of the surgery. Measurements were repeated thrice to determine consistency in pressure measurements, and the mean values of recorded data were determined. The patient's informed consent was obtained prior to the hospitalization and procedures.

Medications and Supplementary Treatments

Tetanus prophylaxis, 50 mg/kg intravenous (IV) ceftriaxone, was administered preoperatively. Postoperative elevation at the heart level was utilized for further pain and swelling relief. Hourly capillary refill time and pain follow-ups were performed. Postoperative treatments also included paracetamol IV, a 50 mg/kg/day maximal dose, was administered to prevent triggered pain and to prevent sympathetic vasoconstrictor activity and ceftriaxone IV, 100 mg/kg, was given twice daily for seven days to treat bacterial infections. Accurate blood glucose and hemoglobin A1c (HbA1c) levels were strictly re-organized and monitored via appropriate diet and insulin administrations in consultation with an endocrinology specialist throughout the treatment and follow-up periods.

Follow-ups and Outcome Measures

The patient was followed-up at the clinic for three days and then was scheduled to return for follow-up assessments on the tenth day, in the first, third, and sixth months, and in the first and second postoperative years. Evaluations were based on monitoring the patient’s muscle and nerve functions, as well as joint inflammation and blood flow status, as described [7-10]: a) capillary refill time (CRT) test to measure blood flow and return in the thenar capillaries [7]; b) visual analogue scale (VAS) score for postoperative assessment of pain intensity [8]; c) two-point discrimination of the affected-unaffected thumbs for sensation testing [9]; d) short version of the Disabilities of the Arm, Shoulder, and Hand questionnaire (QuickDASH)-Turkish and QuickDASH Work Module-Turkish for disability and performance ability, respectively [10]; and e) blood tests: erythrocyte sedimentation rate (ESR) and C-reactive protein (CRP) tests were used to assess any inflammation activities, while blood HbA1c and glucose levels confirmed her comorbidity of diabetes mellitus type 2.

Results

Thenar Compartment Pressure Measurements Using AMAPLT

The thenar compartment pressure was immediately measured by the transducing system of the AMAPLT (Figure 1B), which contained an external disposable fluid-air interface to detect pressure changes. Thus, the pressure in the arterial-posterior blood vessel walls was recorded via a column of fluid between the catheter in the blood vessel and a diaphragm within the transducer. This diaphragm transmitted the changes in pressure to a silicon chip, which detected the signals and displayed it on a monitor as an arterial waveform. After calibration, zero setting, and leveling, the actual arterial pressure readings were displayed on the monitor screen. These recorded readings correlated to thenar and first and second intermetacarpal compartment pressures.

The mean capillary refill time of the thumb was three seconds, mean two-point discrimination differences between the affected and unaffected thumb pulps were 5.1 mm, the mean compartment pressure was 31 mmHg in the thenar compartment, and 23 mmHg at the first and second intermetacarpal compartment, which indicated pressure build-up and a requirement for decompression (Table 1A, 1C). Immediately following the decompression, the thenar and first and second intermetacarpal compartments were simultaneously monitored by the AMAPLT. Final thenar and first and second intercarpal pressures measured 12 and 8 mmHg, respectively (Table 1B, 1D). Postoperatively at the clinical follow-ups, there was significant pain relief during flexion-extension of the thumb MCP and IP joints. This correlated with a decrease of the infectious findings by the establishment of effective blood circulation. At the postoperative Day 3 follow-up, just before the discharge from the hospital, the mean thumb capillary refill time was one second and the mean two-point discrimination differences between the affected left thumb and unaffected right thumb pulps were 2.9 mm revealing the compartment decompressions under AMAPLT monitorization were effective. At the Day 10 follow-up, the patient was completely relieved of preoperative symptoms and had a full range of motion at the MCP and IP joints without infectious clinical findings. Intraoperative microbiological cell cultures revealed methicillin-sensitive Staphylococcus aureus as the cause of infection.

**Table 1:** Measured Compartment Pressure Values Measured compartment pressure values in mmHg before (A, C) and after (B, D) microsurgery. (A) Thenar compartment preoperative; (B) Thenar compartment postoperative; (C) First dorsal intermetacarpal compartment preoperative; (D) First dorsal intermetacarpal compartment postoperative.

Central venous pressure (CVP)	A. Preoperative Thenar Compartment	B. Postoperative Thenar Compartment	C. Preoperative First Intermetacarpal Compartment	D. Postoperative First Intermetacarpal Compartment
Systolic	32 mmHg	13 mmHg	24 mmHg	8 mmHg
Mean	31 mmHg	12 mmHg	23 mmHg	8 mmHg
Diastolic	31 mmHg	11 mmHg	22 mmHg	7 mmHg

Responses to Follow-up Pain and Functional Tests

In the first two postoperative years, follow-up pain and functional evaluations were carried out to assess the progress of the patient’s left-hand restoration. The results of the various tests performed are displayed in Table 2.

**Table 2:** First and Second Year Postoperative Follow-up Pain and Functional Assessment Outcomes and Their Mean Values The mean for each assessment was obtained from at least three determinations. The mean of outcomes for each assessment was obtained by averaging first and second year results. QuickDASH-Turkish: short version of the Disabilities of the Arm, Shoulder, and Hand questionnaire - Turkish version

	Postoperative First Year Outcomes	Postoperative Second Year Outcomes	The Mean of Outcomes
Capillary Refill Time (CRT)	1 second	1 second	1 second
Visual Analogue Scale (VAS) score	4	1	2.5
2-point Discrimination Unaffected-Affected Thumbs	4 mm	1.5 mm	2.75 mm
QuickDASH-Turkish	27.2	11.36	19.28
QuickDASH WorkModule-Turkish	37.5	12.25	25
Hemoglobin A1c (HbA1c)	6.2 mg/dL	5.7 mg/dL	5.95 mg/dL
Blood Glucose	314 mg/dL	265 mg/dL	289.5 mg/dL
Erythrocyte Sedimentation Rate (ESR)	14 mm/hr	22 mm/hr	18 mm/hr
C-reactive protein (CRP)	3 mg/dL	1.12 mg/dL	2.06 mg/dL

Due to the compression build-up prior to surgery, the flexion and abduction of the first metacarpal are typical deformities in TCS. Thus, these measurements were necessary to determine if the left hand was gradually undergoing recovery after the initial microsurgical procedure. The results showed that the CRT was normal (one second), indicating that blood flow in the thumb was adequate. There was a VAS score reduction from four to one (first-year to second-year), which indicated there was still residual pain present in the first year. The muscle and nerves of the thenar space and the first metacarpal had not completely healed by year one. There was, however, remarkable relief by year two, which was consistent with the two-point discrimination unaffected-affected thumb results showing improvement in nerve lesion of the affected areas. Further, the QuickDASH-Turkey and the QuickDASH Work Module-Turkish scores measured muscle function in the affected hand to determine if there was any disability and, if the disability was absent, how well the hand could perform work [10]. The results showed mild difficulty to perform tasks in the first year, but there was a dramatic improvement by year two. Finally, the blood tests showed that any acute phase inflammatory activity still present in the first year was restored to normal by the second year, as revealed by the reduction in the levels of the ESR and CRP test results. There was also an improvement of the patient’s diabetes status, as displayed by the reduction in both HBA1c and blood glucose levels. Favorably, the overall two-year mean follow-up outcomes for both pain and functional assessments were impressive.

## Discussion

Acute compartment syndrome occurs following fractures, crush injuries, or vascular injuries [3]. Without prompt intervention and diagnosis, the pathophysiology of TCS could lead to adverse consequences and complications following such injuries. TCS is initiated by arterial contusion, which results in swelling within a compact space [1]. Artery or venous hemorrhage directly into the compartment increases the space pressure, followed by a marked reduction in arterial blood flow. Consequently, this leads to ischemia and necrosis of the tissue as oxygen requirements are no longer met. The patient experiences paresthesia, characterized by an abnormal prickling sensation, due to damage to the peripheral nerves primarily caused by pressure build-up [1-3, 5].

A recording of compartmental pressures higher than 30 mmHg indicates there is a need to reduce pressure build-up. If immediate treatment does not ensue and pressure rises to 45 mmHg or greater, tissue necrosis and nerve injury could occur within six to 10 hours. Disastrously, irreversible functional loss of the thumb could result in 12 to 24 hours. Thus, the pressing need is to secure a device for immediate and accurate pressure measurements to aid rapid diagnosis in emergency situations and to enable real-time pressure decompression monitoring during microsurgical procedures to ensure pressure release occurs. Disappointingly, several devices have produced inconsistencies in pressure recordings, resulting in a miss of a TCS diagnosis [1-5].

Contrary to pressure measurement errors other investigators have encountered [1-5], our present results show that the modern anesthesia machine arterial pressure transducer (AMAPLT) with the connected slit-modified angiocatheter might serve as an accurate and constant compartment pressure monitoring option for emergencies. It guarantees efficient and precise compartment pressure release; when we substituted the Broody et al. original measurement device or slit catheter with our own, we achieved a comparable outcome [6]. Boody et al. earlier reported the use of a modern anesthesia machine arterial pressure line transducer, which also displayed more accurate pressure information when compared to traditionally specialized compartment measurement devices. Since we did not have access to all the components of this device in our emergency settings, we instead utilized and designed a modified anesthesia machine arterial pressure line transducer as a compartment pressure measuring device, which also exhibited great precision. One limitation is the sophistication of the AMAPLT equipment and handling. Without proper education and hands-on training of doctors, nurses, and technicians, errors could be introduced in the assembly, calibration, and fluid injections, which would ultimately affect the accuracy of compartment pressure measurements.

## Conclusions

In this retrospective case report, we studied the use of a modified compartment device, AMAPLT, which enabled us to rapidly and accurately monitor pressure measurements for diagnosing TCS in a 45-year-old diabetic female, who also had complications of a severe Staphylococcus infection in the injury area. Her TCS was caused by injury through trauma from a fall. Immediately, we performed the only available treatment, which was microsurgery, in order to cut away the thenar fascia and carry out decompression procedures in the left thumb thenar and first interosseous metacarpal compartments, releasing the pressure built-up by the neglected injury. We monitored the decompression process with real-time pressure measurements to guide the pressure release. Additionally, our first- and second-year patient assessment follow-ups were remarkable; the results demonstrated a marked development in patient recovery. The patient was satisfied with the range of motion, absence of pain, and regular hand functions. She was excited about regaining full restoration.

Our present study is the first to report the utilization of this modified device in the immediate diagnosis and effective treatment of TCS. Further randomized controlled trials are needed for various compartment syndrome pressure measurements, including more thenar compartment data, in order to standardize and validate the absolute accuracy of the measurements.
